# Management of renal artery aneurysms: A retrospective study

**DOI:** 10.1177/17085381241263190

**Published:** 2024-07-19

**Authors:** Lisa Vi, Minji Jinny Kim, Naomi Eisenberg, Kong T Tan, Graham Roche-Nagle

**Affiliations:** 1 Division of Vascular Surgery, Peter Munk Cardiac Centre & University Health Network, 7938 University of Toronto, Toronto, ON, Canada; 2 Temerty Faculty of Medicine, 12366 University of Toronto, Toronto, ON, Canada; 3 Division of Interventional Radiology, University Health Network, University of Toronto, Toronto, ON, Canada

**Keywords:** Renal artery aneurysms, endovascular repair, open repair, vascular surgery, aneurysm rupture, vascular disease

## Abstract

**Background:**

Although renal artery aneurysms (RAAs) are rare and often asymptomatic with slow growth, their natural progression and optimal management are not well understood. Treatment recommendations for RAAs do exist; however, they are supported by limited data.

**Methods:**

A retrospective cohort study was conducted to explore the management of patients diagnosed with an RAA at our institution from January 1st, 2013, to December 31st, 2020. Patients were identified through a search of our radiological database, followed by a comprehensive chart review for further assessment. Data collection encompassed patient and aneurysm characteristics, the rationale for initial imaging, treatment, surveillance, and all-cause mortality.

**Results:**

One hundred eighty-five patients were diagnosed with or treated for RAAs at our center during this timeframe, with most aneurysms having been discovered incidentally. Average aneurysm size was 1.40 cm (±0.05). Of those treated, the mean size was 2.38 cm (±0.24). Among aneurysms larger than 3 cm in size, comprising 3.24% of the total cases, 83.3% underwent treatment procedures. Only 20% of women of childbearing age received treatment for their aneurysms. There was one instance of aneurysm rupture, with no associated mortality or significant morbidity.

**Conclusions:**

Our institution’s management of RAAs over the period of the study generally aligned with guidelines. One potential area of improvement is more proactive intervention for women of childbearing age.

## Background

Renal artery aneurysms (RAAs) are uncommon, reportedly occurring in only 0.1% of the general population.^1^ The true incidence has been difficult to ascertain due to their predominantly asymptomatic presentation.^2^ Given their rarity, there is a paucity of the literature on the natural course of RAAs and their clinical significance, and as a result, remain inadequately understood. Few studies have described the natural history of RAAs to be stable or slow growing, ranging from 0.06 to 0.86 mm/year.^2–4^ The risk of rupture for RAAs is also low, estimated at 3–5%, with a rupture rate below 10%.^5^ If left unmanaged, RAAs can lead to hypertension, renal failure, dissection, or most severely, rupture of the renal arterial wall.^4^ The indications for RAA repair thus remain a subject for discussion.

Given the negligible to slow growth rate, low risk of rupture, and the inherent risks associated with treatment,^6–8^ guidelines for the management of RAAs have evolved over time. Historically, guidelines advocated for repair of most visceral artery aneurysms, including renal artery aneurysms larger than two centimeters.^9^ As contemporary research has accumulated, new guidelines were introduced by the Society for Vascular Surgery in 2020. The current recommendations emphasize treatment for patients with renal artery diameters exceeding three centimeters, symptomatic RAAs presenting with symptoms such as flank pain and hematuria, women of childbearing age, and individuals with refractory hypertension and renal stenosis.^10^ There is a persistent lack of long-term contemporary data to substantiate these guidelines, leaving clinicians with uncertainties regarding the optimal management of RAAs, especially given the increasing incidence of incidentally discovered cases attributed to the rise in abdominal imaging over recent decades.^2,6^ Our study examines our institution’s management of RAAs and whether they have aligned with guidelines over time and the impact of these practices on patient outcomes.

## Methods

This was a retrospective study conducted at our local center, which is a quaternary academic center. A radiologic database search from January 1st, 2013, to December 31st, 2020, was used to identify patients found to have an RAA. Ethical approval was obtained through our local ethics review board.

### Data collection

The radiologic database used for this study was a collection of radiologic reports for studies done across the University Health Network (UHN). UHN is a group of hospitals in Toronto, Canada, comprising Toronto General Hospital, Toronto Western Hospital, Princess Margaret Cancer Centre, and Toronto Rehabilitation Institute. This database was searched using the key term “renal artery aneurysm.” Patients who lacked aneurysm diameter measurements in their reports and had no accompanying images for diameter measurements were excluded. Details of the initial radiological examination for each patient were also recorded, including the type of exam (e.g., angiography or CT pelvis), the rationale for ordering the exam, specialty of the ordering provider, and whether follow-up imaging was ordered. Aneurysm characteristics such as diameter, laterality, and morphology were also recorded.

A comprehensive chart review was performed to identify patient characteristics, survival outcomes, and treatment, if any. In accordance with a similar study, women of childbearing age were defined as women aged 18 to 49 years.^11^ Moreover, for individuals who underwent interventions, method of treatment was recorded.

### Statistical analysis

Statistical analysis was conducted using IBM SPSS Statistics (Version 28, IBM, Armonk, New York). This analysis encompassed chi-square testing (or likelihood ratio when appropriate) for categorical variables and T-testing for continuous variables. Results were presented as means, along with corresponding standard errors, for continuous variables, while categorical variables were reported as frequencies and percentages. Significance was defined as having a *p*-value less than 0.05.

## Results

### Patient demographics

Table 1 lists overall demographics. The radiological database identified 185 patients diagnosed with or treated for RAAs at our center. The mean patient age was 67.64 years, with the majority being female (53%). Among the females, 5.40% were of childbearing age, and only 20% of these patients underwent treatment for their RAAs. The laterality of RAAs was predominantly on the left side (55.7%), followed by the right side (35.1%) and bilateral (9.2%).

**Table 1.** Overall demographics for full cohort.

	Mean/Number (*N* = 185)
Patient characteristics	
Age	67.64 yrs (±1.04)
Male	87 (47.0)
Female	98 (53.0)
Number of childbearing females	10 (5.4)
Number of childbearing females treated	2 (1.1)
Abdominal aortic aneurysm	8 (4.3)
Visceral	13 (7.0)
Connective tissue disease	9 (4.9)
Aneurysm characteristics	
Overall aneurysm size	1.40 cm (±0.05)
Average size of those treated	2.38 cm (±0.24)
Right	65 (35.1)
Left	103 (55.7)
Bilateral	17 (9.2)
Intervention	
Treated	19 (10.3)
Endovascular	14 (7.6)
Open	5 (2.7)
Survival	
Rupture	1 (0.5)
Mortality	45 (24.3)
Follow-up	
Vascular surgeon	59 (31.9)
Imaging	55 (29.7)

Numbers are reported as *n* (% of column) for categorical variables or mean (±SE) for continuous variables.

### Treated versus non-treated cohorts

Table 2 lists aneurysm and patient characteristics of treated versus non-treated cohorts. When comparing treated with non-treated cohorts, the mean age was lower (61.05 years vs 68.40 years) and mean aneurysm size was higher (2.38 cm vs 1.28 cm) for treated patients, both of which were found to be statistically significant. Within the subset of aneurysms exceeding 3 cm (3.24%, *n* = 6), a majority (83.3%, *n* = 5) underwent treatment. We noted that 42.2% of RAAs from the non-treated group exhibited calcification, in contrast to only 5.2% of calcified RAAs in the treated group, and this difference was statistically significant. There were no significant differences between treated versus non-treated cohorts for the presence of additional visceral or non-visceral aneurysms, connective tissue disorders, or being of childbearing age for female patients. All cases of abdominal aortic aneurysms were exclusively observed in male patients.

**Table 2.** Aneurysm and patient characteristics of treated versus non-treated cohorts.

	Non-Treated	Treated
	*N* = 166 (89.7)	*N* = 19 (10.3)	*p*-Value
Aneurysm characteristics			
Aneurysm size	1.28 cm (±0.04)	2.38 cm (±0.24)	<0.001*
Right	94 (56.6)	9 (47.3)	0.442
Left	57 (34.3)	8 (42.1)	0.502
Bilateral	15 (9.0)	2 (10.5)	0.831
Calcification	70 (42.2)	1 (5.2)	0.002*
Thrombosed	7 (4.2)	0 (0)	0.213
Dissection	1 (0.6)	0 (0)	0.641
Patient characteristics			
Age	68.40 yrs (±1.06)	61.05 yrs (±3.86)	0.016*
Abdominal aortic aneurysm	7 (4.2)	1 (5.2)	0.836
Another non-visceral aneurysm	3 (1.8)	0 (0)	0.418
Visceral	12 (7.2)	1 (5.2)	0.742
Connective tissue disease	7 (4.2)	3 (15.8)	0.073
Childbearing (females 18–49 years old)	8 (4.8)	2 (10.5)	0.933

Numbers are reported as *n* (% of column) for categorical variables or mean (±SE) for continuous variables. *p < .05.

When we analyzed the annual treatment of RAAs throughout the study duration, a distinct pattern emerged. There was an upward trend in treatment frequency from 2016 to 2017, which was subsequently followed by a gradual decline. During the years 2018 to 2020, not only were fewer patients treated, but also the diameter of the treated aneurysms also appeared to be comparatively smaller when contrasted with earlier periods.

### Endovascular versus open repairs

Only 10.2% of patients received treatment for their RAAs, with the majority being endovascular repairs (73.4%) versus open repairs (26.3%). Those who underwent endovascular repairs tended to be older (mean age of 64.36 years vs 54 years). No statistically significant differences were found in mean aneurysm size or the laterality of RAAs, but both cases of bilateral RAAs in the treated group were managed using an open approach. Furthermore, all three patients with connective tissue disorders received open repairs.

Regarding the types of repairs performed, embolization constituted the majority (10/19 cases, 52.63%), followed by covered stent placement (4/19, 21.05%), excision of aneurysm with a bovine pericardial patch (3/19, 15.79%), nephrectomy (1/19, 5.26%), and renal artery bypass (1/19, 5.26%). Notably, one of the open repairs involved both excision of the aneurysm with a bovine pericardial patch and reimplantation of an upper pole vessel into the renal artery to maintain renal perfusion.

### Reason for exam, ordering provider, and follow-up

Table 3 lists reason for exam, ordering provider, and follow-up for treated and non-treated cohorts. When examining the reasons for initial examination, patients who had explicit indications for treatment of an RAA (89.5% of treated patients) or had mention of RAAs in their summaries (89.5% of treated patients) were more likely to receive treatment, which were both statistically significant. Additionally, patients who received an initial examination due to abdominal symptoms (27.1%) or for cancer-related concerns (38.0%) had RAAs that were incidentally found but ultimately not treated. Vascular surgery was the top specialty that ordered the exams, followed by emergency room physicians and oncologists. Patients whose exams were ordered by vascular surgeons were more likely to receive treatment for their RAAs (75% vs 17%). Though treated patients were more likely to receive follow-up imaging (100% vs 21.7%) or have consultations with vascular surgeons (94.7% vs 24.7%) when compared to non-treated patients, the majority were not followed by a vascular surgeon (68.1%) and did not receive follow-up imaging (70.3%) at our institution.

**Table 3.** Reason for exam, ordering provider, and/or follow-up for treated versus non-treated cohorts.

	Non-Treated	Treated	*p*-Value
	*N* = 166 (89.7)	*N* = 19 (10.3)	
Reason for exam			
Cancer	63 (38.0)	0	<0.001*
Abdominal symptoms	45 (27.1)	0	<0.001*
RAA	28 (16.9)	17 (89.5)	<0.001*
Follow-up on other imaging	10 (6.0)	0	0.135
Postoperative/preoperative	8 (4.8)	0	0.183
Aortic aneurysm/dissection	5 (3.0)	1 (5.2)	0.626
Other visceral aneurysm	2 (1.2)	1 (5.2)	0.274
Transplant workup	1 (0.6)	0	0.641
PAD	2 (1.2)	0	0.509
Trauma	2 (1.2)	0	0.509
Mentioned in summary	62 (37.3)	17 (89.5)	<0.001*
Follow-up			
Vascular surgeon	41 (24.7)	18 (94.7)	<0.001*
Imaging	36 (21.7)	19 (100.0)	<0.001*

Numbers are reported as *n* (% of column) for categorical variables or mean (±SE) for continuous variables. *p < .05.

### Childbearing women

Among the 10 childbearing women diagnosed with an RAA, 80% underwent follow-up imaging (*p* < .001), and 70% had follow-up appointments with a vascular surgeon (*p* = .01). The average aneurysm size in all these women was 1.37 ± 0.05 cm. However, those who received treatment had larger aneurysms, averaging 2.2 ± 0.30 cm.

### Survival outcomes

There was one case of a ruptured RAA which presented as ruptured and was repaired without mortality. The all-cause mortality rate was 24.3%. Deceased patients had a higher mean age (72.02 years) and were less likely to have received follow-up by a vascular surgeon (11.1%) and follow-up imaging (11.1%), both of which were statistically significant.

## Discussion

Given the rarity of RAAs, guidelines have often relied on a limited body of research, resulting in relatively modest recommendations. Over time, as more studies have become available, these guidelines have evolved. The most recent guidelines, published in 2020, have advocated for a more conservative approach to treatment.^10^ Our study focuses on the period before these updated guidelines, when treatment practices might not align with the latest recommendations. We observed that the average treated aneurysm diameter in our cohort stood at 2.38 cm (±0.24). This finding is in keeping with the prevailing guidelines at the time, which recommended intervention for aneurysms exceeding 2 cm in size. Interestingly, our study revealed a gradual decrease in the number of treated aneurysms since 2018, with only one case treated in 2020. This singular patient presented with an RAA and a large endoleak following FEVAR which necessitated intervention. Our findings suggest that even before the official guideline revision in 2020, our institution had begun to adopt a more conservative approach, possibly in response to the increasing recognition of the relatively benign nature of these aneurysms.^2^ When assessing the adoption of new recommendations within the context of updated hypertension guidelines, it became evident that the challenge in embracing new guidelines did not primarily stem from a lack of awareness; rather, it was influenced by practitioners’ readiness to implement guidance that deviated from their established practices.^12^ Our findings suggest that the new RAA guidelines may be readily embraced, given that our institution’s recent practices have already been aligned with a similar conservative approach.

Multiple retrospective studies on the management of RAAs have been conducted, with several emerging over the past decade. In the most recent study conducted by Zhang et al., which investigated the management of RAAs at a single institution between 2015 and 2019, they identified an association between aneurysm diameter exceeding 2.5 cm and the need for treatment (*p* = .006).^11^ In a similar, yet slightly older study by Klausner et al., encompassing RAAs across multiple institutions from 2002 to 2012, the treated aneurysm size was reported as 2.4 cm for asymptomatic cases and 2.3 cm for symptomatic cases.^3^ Further, Wayne et al.’s study, spanning the years 2008 to 2013, revealed that out of fifty-five RAAs, only seven were treated, with 6 of these cases having an aneurysm size of 2.5 cm or greater.^4^ A trend emerged when we examined older studies, which reflected a gradual shift in management over time. In the study by Pfeiffer et al., which examined RAAs between 1980 and 2002, nearly half of the patients underwent treatment when the aneurysm diameter ranged over 1 cm but less than 2 cm.^13^ Similarly, Brownstein et al. found, during the years 1999 to 2016, that the diameter of repaired aneurysms averaged 1.84 +/− 0.55 cm.^14^ In comparison to these earlier studies, our findings align more closely with contemporary research, demonstrating the more conservative approach to RAA management that has evolved over time.

Among the 19 treated aneurysms identified in our study, the majority underwent endovascular treatment (73.4%). When comparing aneurysm diameters between the endovascular and open repair groups, no statistically significant differences were observed. This trend echoed the findings in the study conducted by Zhang et al., which reported that 63% of RAAs were treated by endovascular repair.^11^ The literature also supports the effectiveness of endovascular repair for RAAs. Etezadi et al. investigated the efficacy of endovascular repair in 17 visceral and renal aneurysms and found no clinically significant complications, along with a 98% technical success rate.^15^ Similarly, Buck et al., in their study spanning nationwide RAA repairs from 2000 to 2011, observed an increasing trend in the use of endovascular techniques over the years. However, despite this shift, they noted no significant reduction in operative mortality or the number of open repairs being performed.^16^ Furthermore, in the study conducted by Tsilimparis et al., comparing open and endovascular repair, no differences were detected in long-term renal function between the two approaches, nor were there any clinically significant disparities in reinterventions.^5^ We expect that as endovascular techniques continue to evolve and gain prominence, the rates of endovascular repairs will continue to rise, given the consistent findings of safety and efficacy. This observation is reflected in our study and other contemporary studies, where most cases are treated using endovascular techniques, especially when contrasted with older studies that predominantly relied on reconstructive methods.

Our study revealed that only two of ten (20%) childbearing females were treated throughout the study period. However, current guidelines from the Society for Vascular Surgery (SVS) explicitly recommend treating childbearing females at any aneurysm size.^10^ This discrepancy could, in part, be attributed to the evolution of treatment guidelines, that is, guidelines of the time only recommended treatment for aneurysms larger than 2 cm. Nonetheless, there remained a prevailing consensus to treat childbearing females regardless of size due to the substantial risks associated with maternal and fetal mortality and morbidity in the event of rupture.^17^ While reports of ruptures in pregnant females have been documented in the literature, our study did not identify such cases. We observed only one rupture, which occurred in a male and presented as such. This finding is consistent with many other studies that reported no ruptures over their study periods.^11^ The decision-making process regarding childbearing potential females involves striking a balance between the risk of rupture and the potential harms of intervention. It is important to note that the overall rarity of ruptures, cited at 3–5% in the literature, underscores the complexity of this decision.^1^ Therefore, our institution displayed a notably conservative approach to managing females of childbearing potential compared to the prevailing literature at the time. This emphasizes the importance of adopting updated guidelines that reflect consensus recommendations.

In our study, the presence of calcification of RAAs differed significantly between treated and non-treated groups; only 5.2% of RAAs in the treated group exhibited calcification, compared to 42.2% of RAAs from the non-treated group. In the context of aortic aneurysms, increased vascular calcification is believed to confer protective effects against progressive expansion by stabilizing and stiffening the aneurysmal wall.^18^ Our study did not delve into whether calcification indeed served a protective role in limiting expansion, primarily because a significant portion of our cohort lacked follow-up imaging for expansion analysis. Therefore, further investigation into calcification and the progression of RAAs is warranted.

Considering that the majority of RAAs were incidentally identified, with only 29.7% receiving dedicated follow-up imaging, it is not surprising that only 31.9% of patients were followed by a vascular surgeon during the study period. We observed that better survival was associated with both follow-up by a vascular surgeon and the utilization of follow-up imaging. The decision to undergo treatment did not appear to impact survival. One potential reason may be that follow-up with a vascular surgeon may entail more comprehensive care and surveillance for other comorbidities associated with the etiology of RAAs, such as cardiovascular risk factors and connective tissue diseases.^10,11^ The necessity for ongoing follow-up by a vascular surgeon for RAAs remains somewhat unclear, considering the slow growth rates reported in the literature (ranging from 0.06 to 0.86 mm/year)^2–4^ and the increased size threshold for intervention. There may be potential advantages to involving a vascular surgeon in the care of RAAs, given the underlying etiology of these aneurysms.

## Limitations

Our study had several limitations. Our patient cohort was identified through a radiological database, and subsequent chart reviews were conducted exclusively with access to our institution’s records. Consequently, any instances of vascular surgery follow-up or treatment administered at external facilities remained beyond our scope and could not be captured. Furthermore, cause of death was not captured in this study as most patients did not have this recorded in our institution’s medical records. To address these limitations, future investigations may benefit from utilizing larger provincial administrative databases.

We acknowledge that our study was conducted within the confines of a single institution over a span of 7 years. While our findings provide valuable insights into local practice, they may not be fully representative of broader treatment practices for RAAs. The need for future multi-institutional studies as referred to previously, particularly within the Canadian context, where such investigations are lacking, remains important. Additionally, our study’s timeframe aligns with the initial introduction of new guidelines, precluding a comprehensive assessment of potential shifts in clinical practices that may have arisen in response to these guidelines. Subsequent research should focus on elucidating practice patterns following the adoption of these guidelines to offer a more comprehensive view of evolving trends in RAA management.

## Conclusions

In summary, this single-institution study offers valuable insights into practice patterns that demonstrate consistency with prevailing guidelines throughout the study period. Our findings align with those of contemporary studies investigating the management of RAAs. One area for potential improvement involves more proactive management of women of childbearing age. We note that our study identified only a solitary rupture during the research period, with no associated mortality or morbidity. This observation raises the possibility that a conservative approach to treatment in this demographic is reasonable, as the risks associated with intervention may not outweigh the benefits. The necessity for the involvement of vascular surgeons in the ongoing surveillance of RAAs may warrant revaluation. Given the slow growth and relatively benign nature of these aneurysms, it may be argued that such a level of specialized care may not be imperative. Nonetheless, our findings suggest that such involvement might offer potential survival advantages.
